# Adaptation of *Candida albicans* to specific host environments by gain-of-function mutations in transcription factors

**DOI:** 10.1371/journal.ppat.1012643

**Published:** 2024-11-04

**Authors:** Joachim Morschhäuser

**Affiliations:** Institute of Molecular Infection Biology, University of Würzburg, Würzburg, Germany; University of Georgia, UNITED STATES OF AMERICA

## Abstract

The yeast *Candida albicans* is usually a harmless member of the normal microbiota in healthy persons but is also a major fungal pathogen that can colonize and infect almost every human tissue. A successful adaptation to environmental changes encountered in different host niches requires an appropriate regulation of gene expression. The zinc cluster transcription factors are the largest family of transcriptional regulators in *C*. *albicans* and are involved in the control of virtually all aspects of its biology. Under certain circumstances, mutations in these transcription factors that alter their activity and the expression of their target genes confer a selective advantage, which results in the emergence of phenotypically altered variants that are better adapted to new environmental challenges. This review describes how gain-of-function mutations in different zinc cluster transcription factors enable *C*. *albicans* to overcome antifungal therapy and to successfully establish itself in specific host niches.

## Introduction

**Fig 1:**
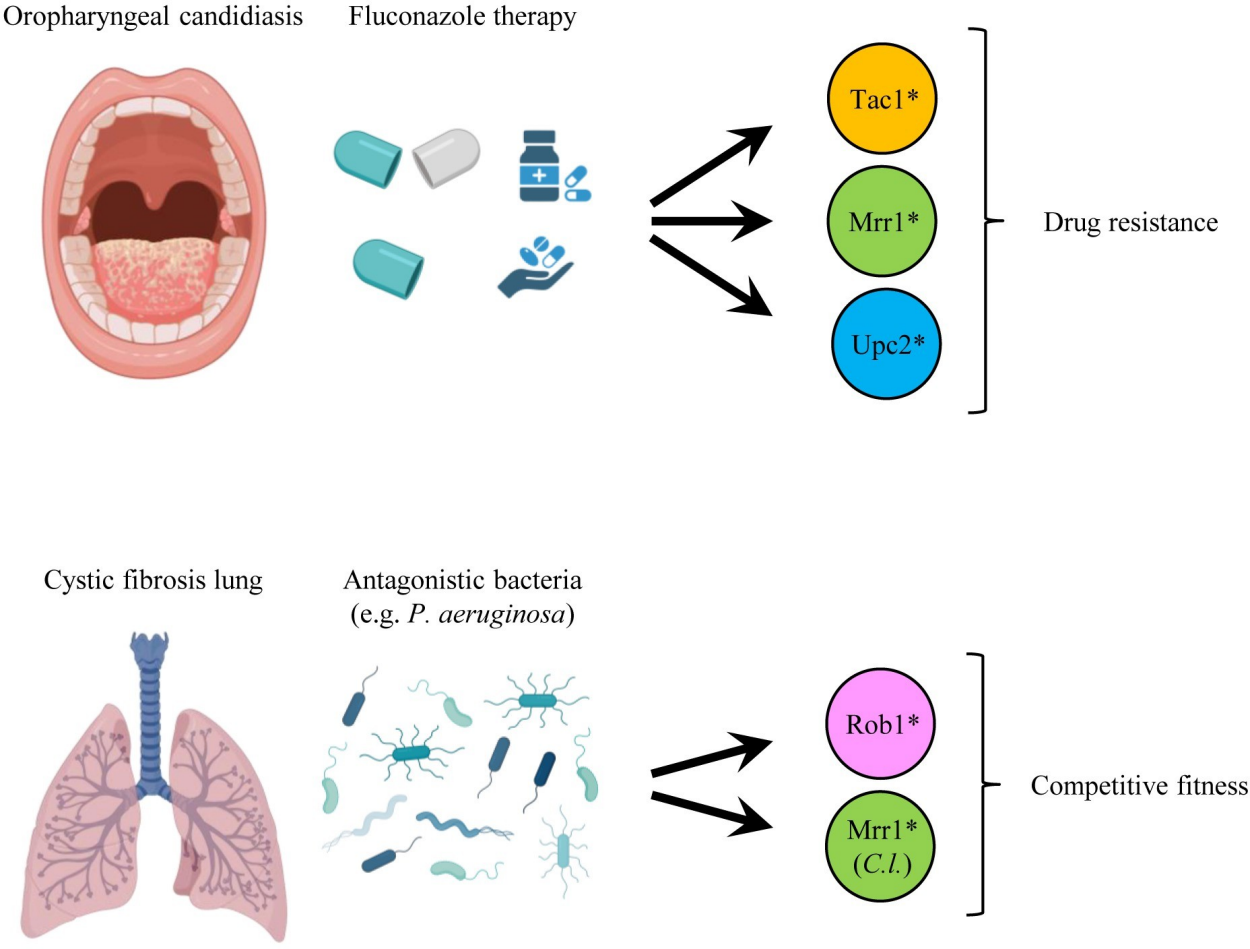
Host environments in which GOF mutations in ZCFs conferred new advantageous phenotypes. Fluconazole treatment of patients with oropharyngeal candidiasis selects for GOF mutations in Mrr1, Tac1, and Upc2, resulting in increased resistance of *C*. *albicans* and other *Candida* species to the drug. In cystic fibrosis patients who are colonized by *Candida* species and bacteria such as *P*. *aeruginosa*, GOF mutations in the ZCFs Rob1 or Mrr1 result in increased filamentous growth of *C*. *albicans* and phenazine resistance in *C*. *lusitaniae*, respectively, which enhances their fitness and survival in the presence of these competing bacteria. Figure created with Biorender.

The regulation of gene expression is crucial for the ability of cells to adapt to changes in their environment, but it is also an important determinant in the development of organisms and the evolution of species [1]. Transcription factors (TFs) play a central role in gene regulation, acting either as activators or repressors of gene expression and thereby enabling appropriate responses to external signals or alterations in intracellular conditions. A family of TFs that are almost exclusively found in the fungal kingdom are the zinc cluster transcription factors (ZCFs), also known as zinc binuclear cluster or Zn(II)_2_Cys_6_ proteins [2]. The ZCFs contain 6 cysteines in their DNA-binding domain that coordinate 2 zinc atoms, which is required for proper folding and function. They usually bind as homo- or heterodimers to DNA sequences containing CGG triplets, which can occur as repeats in direct, inverted, or everted orientation with variable spacing. In most cases, the DNA-binding domain is located at the N-terminus and separated from a C-terminal transcription activation domain by a large regulatory region that controls the activity of the TF. The diploid pathogenic yeast *Candida albicans* contains 82 (putative) ZCFs, as defined by the extended signature motif CX_2_CX_6_CX_5-24_CX_2_CX_6-9_C, making them the largest family of TFs in this species [3,4]. They are involved in the regulation of virtually all cellular activities, including nutrient utilization, cell wall architecture, stress resistance, the transition from yeast to filamentous growth, white-opaque switching, and mating [5–36]. While proper regulation of ZCFs (and other TFs) is important to reversibly adjust gene expression in response to fluctuating environmental conditions, mutations that permanently alter the activity of a ZCF and the expression of its target genes can be advantageous in certain situations. This review describes how such mutations give rise to phenotypically altered *C*. *albicans* subpopulations that are better adapted to new environmental challenges and conditions encountered in specific host niches (see Fig 1 and Table 1 for an overview).

**Table 1:** GOF mutations in ZCFs conferring new phenotypes.

ZCF	Relevant target genes	Selection	Phenotype
Tac1	*CDR1*, *CDR2*	Fluconazole therapy (clinical isolates)	Fluconazole resistance
	*CDR1*, *CDR2* (presumably)	In vitro selection for inhibitor resistance	Beauvericin resistance
Mrr1	*MDR1* and additional genes	Fluconazole therapy (clinical isolates)	Resistance to fluconazole and histatin 5
	*MDR1*	Cystic fibrosis lung environment (*C*. *lusitaniae*)	Resistance to phenazine toxins secreted by *P*. *aeruginosa*
Upc2	*ERG11* and other *ERG* genes	Fluconazole therapy (clinical isolates)	Fluconazole resistance
Zcf29	Unknown	*In vitro* selection for inhibitor resistance	Resistance to beauvericin and manogepix
Mrr2	*CDR1*	Artificial activation	Fluconazole resistance
Znc1	*CDR1*	Artificial activation	Fluconazole resistance
	*RTA3*	Artificial activation	Miltefosine resistance
Stb5	*YOR1*	Artificial activation	Resistance to oligomycin and beauvericin
Rob1	Filamentation genes	Cystic fibrosis lung environment	Increased filamentation, resistance to inhibition by *P*. *aeruginosa*
	Filamentation genes	*In vitro* selection for inhibitor resistance	Resistance to filamentation inhibition by 1-acetyl-β-carboline

## Mutations in ZCFs conferring antifungal drug resistance

In *C*. *albicans*, ZCFs became a focus of attention when their key role in the development of resistance against the antifungal drug fluconazole was revealed. Fluconazole is widely used to treat infections by *C*. *albicans* and other *Candida* species. The drug inhibits the enzyme sterol 14α-demethylase (Erg11) and thereby impedes the biosynthesis of ergosterol, the main sterol in fungal cell membranes. Early work showed that, compared to fluconazole-susceptible isolates from the same patients, many fluconazole-resistant clinical *C*. *albicans* isolates overexpressed genes encoding multidrug efflux pumps, the ATP-binding cassette (ABC) transporters Cdr1 and Cdr2 and the major facilitator Mdr1, and deletion of these genes from such strains demonstrated that these efflux pumps mediated drug resistance [37–50]. Yet, for a long time, the molecular basis of the constitutive *CDR1*/*CDR2* and *MDR1* up-regulation in these isolates remained unknown. Two observations enabled the identification of the transcription factor that regulates *CDR1*/*CDR2* expression in response to certain inducing chemicals (such as the drug fluphenazine) and is responsible for their overexpression in fluconazole-resistant strains. A typical ZCF binding sequence was identified in the upstream region of both *CDR1* and *CDR2* and found to be required for their up-regulation, indicating that a ZCF mediates their expression [51]. Furthermore, it was found that fluconazole resistance is frequently associated with homozygosity at the mating type locus (*MTL*), although *MTL* homozygosity itself did not result in fluconazole resistance, suggesting that loss of heterozygosity (LOH) for a gene in the vicinity of *MTL* might be responsible for drug resistance [52,53]. Coste and colleagues therefore tested three genes encoding putative ZCFs that were located near *MTL* and found that deletion of one of them, designated *TAC1* (for transcriptional activator of *CDR* genes), resulted in the loss of *CDR1*/*CDR2* induction by fluphenazine [54]. Further work showed that *CDR1*/*CDR2-*overexpressing isolates contained homozygous gain-of-function (GOF) mutations in *TAC1* that rendered the transcription factor constitutively active in the absence of an inducing signal. When introduced into a drug-susceptible laboratory strain, these mutations resulted in constitutive *CDR1*/*CDR2* up-regulation and increased fluconazole resistance, with a much stronger effect when only a mutated allele was present, explaining the LOH in drug-resistant clinical isolates [54,55]. Two other Tac1 target genes, *PDR16* and *RTA3*, which function in membrane lipid composition, have also been shown to contribute to the fluconazole resistance of strains with Tac1 GOF mutations [56,57]. GOF mutations in *TAC1* have subsequently been found in many fluconazole-resistant clinical *C*. *albicans* isolates and also in *TAC1* orthologs of *Candida parapsilosis* and *Candida auris* and shown to cause increased drug resistance [58–69].

The transcription factor that mediates *MDR1* up-regulation was identified by comparing the genome-wide transcription profiles of 3 different *MDR1*-overexpressing fluconazole-resistant *C*. *albicans* isolates and genetically related fluconazole-susceptible isolates from the same patients. Among a core set of genes that were commonly up-regulated in all 3 resistant isolates, one encoded a predicted ZCF, and deletion of this gene, termed *MRR1* (for multidrug resistance regulator), in resistant isolates abolished *MDR1* expression and resulted in increased fluconazole susceptibility [70]. The resistant isolates had acquired GOF mutations in one of the *MRR1* alleles and become homozygous for the mutated allele. The introduction of these mutations into the fluconazole-susceptible *C*. *albicans* reference strain SC5314 caused constitutive *MDR1* overexpression and increased drug resistance, with a stronger effect when no wild-type allele was present [70]. A follow-up study found that all tested fluconazole-resistant *C*. *albicans* isolates that strongly overexpressed *MDR1* contained GOF mutations in *MRR1*, and almost all of them had become homozygous for the mutated allele [71]. Strains that contained a mutated and a wild-type *MRR1* allele exhibited intermediate resistance levels, and both the increased copy number of mutated alleles and the loss of the wild-type allele were shown to contribute to the increased fluconazole resistance of strains that had become homozygous for *MRR1* GOF mutations [70–73]. Deletion of *MRR1* reduced fluconazole resistance more than deletion of *MDR1* [50,70], and hyperactive forms of Mrr1 could still confer increased fluconazole resistance, albeit less efficiently, in the absence of *MDR1* [73]. However, which of the other Mrr1 target genes contribute to fluconazole resistance has not been revealed so far [4,74,75]. GOF mutations in *MRR1* have since been found as a cause of drug resistance in many additional *C*. *albicans* isolates and other medically important *Candida* species [62,76–86].

Like its ortholog in the model yeast *Saccharomyces cerevisiae*, the ZCF Upc2 up-regulates the expression of ergosterol biosynthesis (*ERG*) genes in response to ergosterol depletion in *C*. *albicans* [20,29]. Dunkel and colleagues therefore sequenced the *UPC2* alleles of a fluconazole-susceptible and a genetically related fluconazole-resistant *C*. *albicans* isolate from an AIDS patient in which resistance correlated with constitutive *ERG* gene overexpression in the absence of the drug [87]. The resistant isolate had acquired a GOF mutation in one of its *UPC2* alleles, and introduction of the mutated allele into strain SC5314 resulted in *ERG* gene up-regulation and increased fluconazole resistance regardless of the presence or absence of a wild-type allele [87]. Introduction of the mutation into the second wild-type *UPC2* allele caused a modest further increase in resistance, indicating that homozygosity for a hyperactive *UPC2* allele could be advantageous under fluconazole therapy [88]. Indeed, Heilmann and colleagues found that a fluconazole-resistant isolate that exhibited increased *ERG11* mRNA levels compared with a susceptible isolate from the same patient had become homozygous for a mutated *UPC2* allele that was already present in the heterozygous susceptible isolate [88]. In this case, too, introduction of the GOF mutation into one or both *UPC2* alleles of strain SC5314 resulted in a stepwise increase in fluconazole resistance coupled with increased *ERG11* expression [88]. GOF mutations in *UPC2*, either heterozygous or homozygous, have subsequently been found in many other *ERG11*-overexpressing *C*. *albicans* isolates and shown to confer increased fluconazole resistance [85,89–91], although *ERG11* overexpression can also be achieved by gene amplification [92]. Constitutive activity of Upc2 has a weaker effect on fluconazole resistance than GOF mutations in Tac1 or Mrr1 [72]. This is likely because the ergosterol depletion caused by the drug also activates wild-type Upc2 and up-regulates *ERG* gene expression, such that the constitutive *ERG* gene overexpression caused by Upc2 GOF mutations confers only a moderate advantage in the presence of the antifungal.

## Experimental discovery of advantageous GOF mutations in ZCFs

The potential for GOF mutations in ZCFs to confer novel advantageous phenotypes has also been uncovered by in vitro selection and by engineering strains expressing artificially activated ZCFs. Shekhar-Guturja and colleagues grew *C*. *albicans* in the presence of beauvericin, a compound that sensitizes the cells to fluconazole by inhibiting the efflux pump Cdr1, to select for beauvericin-resistant mutants [93]. Several mutants with a GOF mutation in Tac1 were obtained, demonstrating that selection for Tac1 hyperactivity by beauvericin treatment can simultaneously result in fluconazole resistance and vice versa. Other resistant mutants contained different GOF mutations in the uncharacterized Zcf29, which were confirmed to be responsible for beauvericin resistance. One of the Zcf29 GOF mutations was also identified in a separate study after selection for resistance to the new antifungal manogepix [94]. Transcriptional profiling and DNA-binding studies revealed Zcf29 target genes, including several genes encoding transporters, but the gene(s) responsible for beauvericin resistance was not established (*YOR1*, which encodes a transporter that the authors showed to mediate beauvericin resistance, was not among the Zcf29 target genes) [93].

The functions of some uncharacterized ZCFs and the potential of acquiring new advantageous phenotypes by activating mutations in these TFs have also been uncovered in a targeted approach when it was observed that the addition of a HA tag to the C-terminus of Upc2 and Mrr1 resulted in their constitutive activity, which was further increased by the addition of the heterologous Gal4 activation domain [4,73,95]. Schillig and Morschhäuser used this strategy to express all *C*. *albicans* ZCFs in such a potentially hyperactive form and revealed the target genes of some that conferred an altered phenotype by transcriptional profiling [4]. This systematic investigation identified Mrr2 and Znc1 as novel regulators of *CDR1* expression and showed that activated forms of these ZCFs conferred Cdr1-dependent fluconazole resistance. Stb5 was identified as a regulator of *YOR1* expression that could confer resistance to the Yor1 substrates oligomycin and beauvericin [96], and Znc1 was also found to regulate *RTA3* expression and thereby mediate wild-type tolerance to the drug miltefosine [27]. While natural GOF mutations in these ZCFs have not been reported so far, such activating mutations may be discovered in isolates obtained from environments in which they confer a selective advantage.

## GOF mutations in ZCFs conferring increased fitness in specific host niches

Rob1 is a ZCF that contributes to filamentation and is required for biofilm formation and virulence of *C*. *albicans* [97–103]. The reference strain SC5314 contains a mutation in one of its *ROB1* alleles that results in a P946S substitution, which increased filamentation and biofilm formation under certain conditions but also reduced colonization in a mouse model of oropharyngeal candidiasis due to a stronger inflammatory host response [97]. An additional Rob1 mutation (L672V) that caused hyperfilamentation appeared in derivatives of strain SC5314 after screening for resistance to 1-acetyl-β-carboline, a compound that inhibits filamentation of *C*. *albicans* [104]. Intriguingly, a GOF mutation in Rob1 (G299E) has recently also been found in a subpopulation of *C*. *albicans* isolates from a cystic fibrosis (CF) patient [105]. The isolates were heterozygous for the mutation and exhibited increased filamentation and biofilm formation compared with isolates from the same patient that did not contain the mutation. Introduction of the G299E mutation into one of the latter isolates similarly increased filamentation, expression of hypha-associated genes, and biofilm formation, confirming that it was a driver of these phenotypes. The mutants also displayed enhanced survival in mixed biofilms with *Pseudomonas aeruginosa*, which colonized the lungs of the same patient, and were resistant to *P*. *aeruginosa*-mediated inhibition of filamentous growth. These findings provided evidence that the *ROB1* GOF mutation improved *C*. *albicans* adaptation to the CF lung environment [105]. Another intriguing example of host adaptation came from a different CF patient whose lung was chronically infected with *Candida lusitaniae* [80]. Genome analysis of a large number of isolates from this patient demonstrated the presence of diverse subpopulations of the same strain, many of which contained different GOF mutations in Mrr1. Like *C*. *albicans* isolates with a hyperactive Mrr1, these isolates overexpressed *MDR1* and exhibited increased resistance to fluconazole, although the patient had not been treated with the drug before the isolates were recovered. Mrr1-mediated *MDR1* overexpression also protected the cells from phenazine toxins, which are produced by *P*. *aeruginosa* in the CF lung, suggesting that this may have been the driving force that selected for the multiple acquisition of Mrr1 GOF mutations in this environment. Furthermore, the strain persisted during a subsequent fluconazole therapy, indicating that the selection for Mrr1 GOF mutations by phenazines or other host and bacterial factors conferred an additional advantage when the population faced the new challenge of fluconazole treatment [80,106]. Interestingly, some of the isolates also contained secondary mutations in *MRR1* that either suppressed the constitutive activity of the transcription factor or inactivated it, resulting in the loss of fluconazole resistance. [107]. This may have occurred to revert the fitness costs caused by the deregulated expression of its target genes, which have also been demonstrated for *C*. *albicans* strains containing GOF mutations in Mrr1, Tac1, or Upc2 [72]. Demers and colleagues showed that *C*. *lusitaniae* (and also *C*. *albicans* and *C*. *dubliniensis*) strains with Mrr1 GOF mutations exhibited increased sensitivity to H_2_O_2_, a host-produced defense molecule against pathogens, which was reverted by the secondary mutations found in some of the *C*. *lusitaniae* isolates [107]. Therefore, a trade-off between fluconazole and H_2_O_2_ resistance resulted in a mixed population of cells with GOF mutations in Mrr1 (fluconazole-resistant, H_2_O_2_-hypersensitve) and derivatives with secondary mutations in *MRR1* that suppressed Mrr1 hyperactivity (fluconazole-sensitive, wild-type H_2_O_2_ tolerance). In *C*. *albicans* strains with Mrr1 GOF mutations, the overexpression of *MDR1* and other Mrr1 target genes was found to confer increased resistance to histatin 5, an antimicrobial peptide that is produced in the oral cavity of humans and kills *C*. *albicans* [108]. Of note, while most Mrr1 GOF mutations have been found in *C*. *albicans* isolates from patients with oropharyngeal candidiasis, isolates containing the activating mutations were recovered only under fluconazole therapy. This indicates that fitness costs caused by dysregulated Mrr1 activity outweigh any advantage gained by increased histatin 5 resistance in the absence of selective pressure by drug treatment in this environment [72].

## Genome alterations involving ZCFs

**Fig 2:**
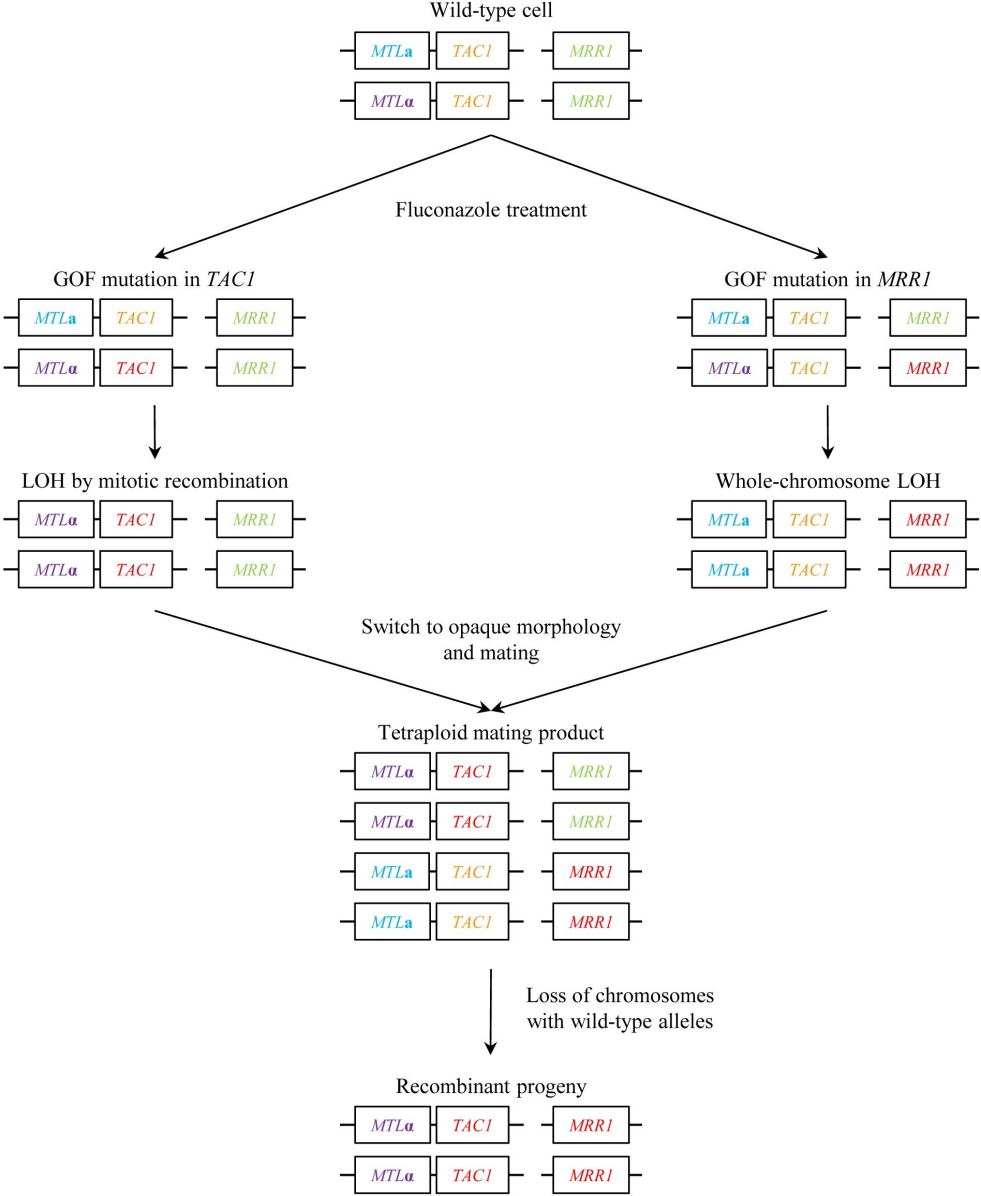
Generation of fluconazole-resistant *C*. *albicans* strains by GOF mutations in ZCFs and drug-induced genome rearrangements that result in concomitant LOH for the resistance mutation and the mating type locus *MTL*. The latter enables the cells to switch to the mating-competent opaque morphology, mate with other *MTL*-homozygous cells containing a different resistance mutation, and produce recombinant progeny containing the resistance genes from both parents. See text for further explanations.

As explained above, GOF mutations in *TAC1*, *MRR1*, and *UPC2* that confer increased fluconazole resistance have a stronger effect when the cells are homozygous for the mutated alleles, and this is also the case for resistance mutations in *ERG11*, encoding the drug target [72]. The recombination events that result in LOH can be local and affect only the *ZCF* gene itself or can involve large regions on the same chromosome arm and even whole chromosomes [58,71]. Homozygosity for a whole chromosome occurs by duplication of one of the 2 homologs and loss of the other homolog and involves a transient aneuploidy. Fluconazole increases the frequency of LOH by mitotic recombination and also causes chromosome segregation defects that result in aneuploidies [109,110]. The drug therefore not only selects for resistance mutations but also promotes genome rearrangements that further augment drug resistance. While LOH for other affected genes may not contribute to drug resistance, it can have additional phenotypic consequences. A prominent example is the already mentioned LOH for the mating type locus, which is frequently seen in strains that are homozygous for resistance mutations in *TAC1* and *ERG11*, both of which are physically linked to *MTL* on the left arm of chromosome 5. *MTL* homozygosity confers upon the cells the ability to switch to the mating-competent opaque morphology, mate with cells of the opposite mating type, and produce recombinant progeny in a parasexual cycle [111,112]. Increases in the rate of *MTL* homozygosity are not necessarily restricted to fluconazole-resistant strains with mutations in *ERG11* or *TAC1*, as fluconazole-induced chromosome missegregation may affect several chromosomes at the same time. Indeed, simultaneous LOH for both *MTL* and a GOF mutation in *MRR1*, which is located on chromosome 3, was observed after growing heterozygous cells in the presence of fluconazole to select for increased drug resistance [113]. *MTL* homozygous cells with different resistance mutations could mate with one another and produce recombinant progeny that contained the resistance mutations of both parents and exhibited further increased resistance after the subsequent loss of the chromosomes with the wild-type alleles [113]. While this sequence of events was observed in controlled laboratory experiments, drug-induced mating competence and parasexual recombination may help explain how highly resistant *C*. *albicans* strains that display combinations of different resistance mechanisms evolve in patients under fluconazole therapy [114] (Fig 2).

## Acquisition of novel functions

Besides enabling short-term adaptations to changes in environmental conditions, transcription factors can also acquire novel functions during the evolution of species. This can include regulating different sets of target genes or controlling their expression in response to different stimuli [115,116]. This is also the case for some zinc cluster transcription factors. For example, in the model yeast *S*. *cerevisiae*, the ZCF Gal4 induces the expression of genes involved in galactose utilization when this sugar is present instead of glucose, but this is not the case in *C*. *albicans*. While Gal4 binds to the same *cis*-regulatory DNA sequence (CGGN_11_CCG) in both species, this motif has been lost from *C*. *albicans GAL* genes, and Gal4 itself is not regulated by galactose availability. Instead, the Gal4 binding motif is present at other genes in *C*. *albicans*, including glycolysis genes that are coregulated by Gal4 and the bHLH transcription factor Tye7 [6,117]. Similarly, the ZCF Ppr1, which induces the expression of uracil biosynthesis genes upon uracil depletion in *S*. *cerevisiae*, promotes expression of genes required for purine catabolism in *C*. *albicans*, although it binds to the same sequence motif (CGGN_6_CCG), which is contained in the *URA* genes in *S*. *cerevisiae* and in purine catabolism genes in *C*. *albicans* [31]. If and how *C*. *albicans* profits from these differences in Gal4 and Ppr1 function is not known, but this may have increased its adaptation to life in a mammalian host. Another example is the ZCF Lys14, which regulates lysine biosynthesis genes in *S*. *cerevisiae*. In *C*. *albicans*, the *LYS14* gene underwent successive gene duplications to produce 4 paralogs, Lys14, Lys142, Lys143, and Lys144, none of which regulates lysine biosynthesis genes. Instead, all 4 paralogs have acquired distinct functions by binding to different sets of genes that are important for growth and survival in specific host niches [118,119].

The potential for evolving increased adaptation to new environmental challenges by mutations in transcription factors that enable them to respond to a normally noninducing signal or regulate additional genes has also become evident by studying phenotypes conferred by genetically engineered hybrid ZCFs in *C*. *albicans* [120]. As noted above, the ZCFs Tac1 and Mrr1 regulate genes encoding multidrug efflux pumps that confer resistance to fluconazole. However, they do not induce the expression of their target genes in response to fluconazole, and strong overexpression of *CDR1*/*CDR2* and *MDR1* has only been observed in strains containing GOF mutations that result in constitutive activity of Tac1 and Mrr1, respectively. In contrast, Upc2 induces the expression of *ERG* genes in the presence of fluconazole and thereby enables the cells to up-regulate ergosterol biosynthesis and grow at subinhibitory drug concentrations, as Upc2 senses ergosterol levels and is activated by the depletion of ergosterol [121]. Hybrid transcription factors in which the DNA-binding domain of Upc2 was replaced by those of Tac1 or Mrr1 induced the expression of the efflux pump genes in response to fluconazole and therefore conferred increased drug resistance [120]. Although no natural GOF mutations with a similar effect have been reported yet, they may well be uncovered by systematic inspection of the ever-increasing number of available genome sequences of *C*. *albicans* strains obtained from different sources. Evaluation of these genomes may identify novel mutations in ZCFs conferring drug resistance and could also uncover mutations in other transcription factors in which the ability to respond to new signals may be advantageous in specific host niches.

## Adaptation to specific host environments by mutations in other transcription factors

While this review focuses on the zinc cluster transcription factors, mutations in other transcriptional regulators have also been linked to *C*. *albicans* adaptation to specific host niches. In some cases, it is the loss of a transcription factor that provides an advantage in a certain environment. A striking example is again the microevolution of *C*. *albicans* during colonization of the lungs in CF patients. In a study by Kim and colleagues, several patients harbored subpopulations of *C*. *albicans* that exhibited a constitutively filamentous phenotype due to loss-of-function mutations in both alleles of the *NRG1* gene, which encodes a C_2_H_2_ zinc finger transcription factor that represses filamentous growth [122]. These isolates were resistant against the filamentation-repressing activities of bacteria (*P*. *aeruginosa* or *Burkholderia multivorans*) that co-colonized the lungs of the same patients, suggesting that these bacteria may have selected for the loss of Nrg1 function as a common mechanism for adaption to the CF lung environment [122]. This scenario is further supported by the recent identification of a GOF mutation in *ROB1* in another *C*. *albicans* isolate from a CF patient, which produced a similar phenotype and conferred resistance to *P*. *aeruginosa*-mediated inhibition of filamentous growth (see above). Conversely, in some host environments, cells that have lost the ability to grow as hyphae have a selective advantage and outcompete wild-type cells. The basic helix-loop-helix (bHLH) transcription factor Efg1 regulates filamentous growth (as well as other cellular processes), and genetically engineered *efg1*Δ mutants are strongly attenuated for virulence in a mouse model of systemic candidiasis [123]. However, during gastrointestinal (GI) colonization of mice, cells lacking Efg1 can outcompete wild-type cells [124–126]. Notably, this is seen only in mice treated with antibiotics to reduce the competing bacterial microbiota. In the absence of antibiotic treatment, the ability to grow as hyphae is important, as wild-type mice outcompete *efg1* mutants [127,128]. Loss of Efg1 may also be advantageous under specific circumstances in human hosts. Hirakawa and colleagues described a nonfilamentous clinical isolate with a homozygous loss-of-function mutation in *EFG1*, which exhibited decreased virulence but, in line with the studies mentioned above, increased fitness during GI colonization compared with a derivative with a restored *EFG1* copy [129]. Somewhat counterintuitively, the strain was isolated from a patient with a bloodstream infection, where the ability to grow as hyphae is supposed to be critical for a successful infection, and the authors hypothesized that the *efg1* mutation may provide a selective advantage only in an immunosuppressed host. A subsequent study found that many natural isolates, both commensal and clinical, contained only one functional *EFG1* allele and could become homozygous *efg1* null mutants at high frequency, which then outcompeted their parents in mouse colonization and infection models [124]. Filamentation-defective mutants exhibiting increased fitness in the mouse gut were also isolated after serially passaging the wild-type strain SC5314 in antibiotic-treated mice via fecal transfer [128]. Most of the evolved strains contained homozygous loss-of-function mutations in *FLO8*, encoding another transcription factor that is required for hyphal growth and virulence in the mouse model of disseminated candidiasis [130]. The increased fitness in the gut colonization model was reproduced in genetically engineered *flo8*Δ mutants, and introduction of a wild-type *FLO8* copy into the evolved *flo8* mutants restored filamentation and decreased their fitness [128]. Both *efg1* and *flo8* mutants caused less cellular damage, which may increase their ability to colonize the intestine [128]. These examples further illustrate that alterations in transcription factors can result in genetic variants that are better adapted to specific host environments.

## Concluding remarks

The examples presented in this review article show that mutations that alter the activity of ZCFs confer new phenotypes that can be advantageous for *C*. *albicans* under certain conditions encountered in the human host and result in the expansion of the mutants in the population. Most of the described mutations result in drug resistance and are selected for during antifungal therapy of patients with *Candida* infections (GOF mutations in Tac1, Mrr1, and Upc2), but they can also increase fitness in certain host niches, such as the CF lung in which fungal cells have to compete with antagonistic bacteria (Rob1, Mrr1). In most cases, it is still not known how inducing conditions increase ZCF activity and how these are mimicked by GOF mutations, and these questions are important areas for future research. Such GOF mutations occur in the regulatory as well as the activation domains of Tac1 and Mrr1, indicating that the ZCFs can be activated in various ways. It is conceivable that GOF mutations can occur in most if not all ZCFs, and they may be uncovered by appropriate in vitro selection conditions, as has been shown for Zcf29. Whether similar mutations in other ZCFs will be found in natural *C*. *albicans* populations colonizing and infecting humans depends on their ability to confer a selective advantage in a specific host niche or under certain physiological conditions. This will be another fascinating field of investigation that can harness the knowledge of phenotypes conferred by in vitro selected GOF mutations and artificially activated ZCFs.
